# The HDAC3 enzymatic activity regulates skeletal muscle fuel metabolism

**DOI:** 10.1093/jmcb/mjy066

**Published:** 2018-11-14

**Authors:** Shiyang Song, Yefei Wen, Hui Tong, Emanuele Loro, Yingyun Gong, Jidong Liu, Sungguan Hong, Lei Li, Tejvir S Khurana, Maoping Chu, Zheng Sun

**Affiliations:** 1 Children's Heart Center, The Second Affiliated Hospital and Yuying Children's Hospital, Institute of Cardiovascular Development and Translational Medicine, Wenzhou, China; 2 Division of Endocrinology, Diabetes, and Metabolism, Department of Medicine, Baylor College of Medicine, Houston, TX, USA; 3 Department of Physiology and Pennsylvania Muscle Institute, Perelman School of Medicine, University of Pennsylvania, Philadelphia, PA, USA; 4 Department of Chemistry, Chung-Ang University, Seoul, Korea; 5 Department of Molecular and Cellular Biology, Baylor College of Medicine, Houston, TX, USA

**Keywords:** HDAC, muscle metabolism, nuclear receptor corepressor, histone deacetylation

## Abstract

Histone deacetylase 3 (HDAC3) is a major HDAC, whose enzymatic activity is targeted by small molecule inhibitors for treating a variety of conditions. However, its enzymatic activity is largely dispensable for its function in embryonic development and hepatic lipid metabolism. HDAC3 plays a pivotal role in regulating muscle fuel metabolism and contractile function. Here, we address whether these muscular functions of HDAC3 require its enzymatic activity. By mutating the NCoR/SMRT corepressors in a knock-in mouse model named NS-DADm, we ablated the enzymatic activity of HDAC3 without affecting its protein levels. Compared to the control mice, skeletal muscles from NS-DADm mice showed lower force generation, enhanced fatigue resistance, enhanced fatty acid oxidation, reduced glucose uptake during exercise, upregulated expression of metabolic genes involved in branched-chain amino acids catabolism, and reduced muscle mass during aging, without changes in the muscle fiber-type composition or mitochondrial protein content. These muscular phenotypes are similar to those observed in the HDAC3-depleted skeletal muscles, which demonstrates that, unlike that in the liver or embryonic development, the metabolic function of HDAC3 in skeletal muscles requires its enzymatic activity. These results suggest that drugs specifically targeting HDAC3 enzyme activity could be developed and tested to modulate muscle energy metabolism and exercise performance.

## Introduction

Small molecule compounds that inhibit HDAC enzymatic activities are known as HDAC inhibitors (HDIs), which emerge as a promising class of drugs for a variety of conditions including cancer, immune diseases, metabolic disorders, and neurological diseases (Christensen et al., 2011; Falkenberg and Johnstone, 2014). Hundreds of ongoing clinical trials involve HDIs with several compounds already approved for treating lymphoma or myeloma in clinic (Falkenberg and Johnstone, 2014). Understanding the physiological function of each individual HDAC and whether their function is dependent on the enzymatic activity is important for the development and application of HDIs.

Mammalian HDACs are classified into four classes based on their sequence homology and catalytic mechanisms. Classes I, II, and IV are the classical zinc-dependent HDACs, while class III are sirtuins dependent on nicotinamide adenine dinucleotide (NAD) (Yang and Seto, 2008). Class I HDACs, including HDAC1, HDAC2, and HDAC3, are located primarily in the nucleus and exist in multiprotein transcriptional coregulator complexes. While HDAC1/HDAC2 exist in the NuRD, Sin3, and CoREST complexes, HDAC3 exist in the NCoR/SMRT complex (Yang and Seto, 2008). Nuclear receptor corepressor (NCoR) and silencing mediator for retinoid and thyroid receptor (SMRT) are two homologous corepressors that can interact with multiple nuclear receptors or other transcription factors (Perissi et al., 2010). Class IIa HDACs (HDAC4, HDAC5, HDAC7, and HDAC9) are also found in the NCoR/SMRT complex but lack intrinsic catalytic activities due to a missense mutation in the catalytic pocket (Lahm et al., 2007). Therefore, HDAC3 is the enzyme that confers deacetylase catalytic activity to the NCoR/SMRT complex and class IIa HDACs (Fischle et al., 2002). Interestingly, purified HDAC3 protein does not have catalytic activity. Instead, its catalytic activity relies on the stable association with the deacetylase activation domain (DAD) located on the N-terminus of NCoR and SMRT (Guenther et al., 2001). Binding with the DAD causes a conformational change in the HDAC3 protein, which exposes the catalytic channel and is required for the enzymatic activity (Watson et al., 2012).

HDAC3 plays important roles in physiological homeostasis in multiple tissues. HDAC3 inhibition is considered to be of therapeutic benefit against cancer, immune diseases, metabolic disorders, and neurological diseases (Cao et al., 2018). We and others previously showed that the enzymatic activity of HDAC3 is not required for some of its functions *in vivo* (Sun et al., 2013; You et al., 2013; Lewandowski et al., 2015; Poleshko et al., 2017). Whole-body knock-in mutations in DADs of both NCoR and SMRT (NS-DADm) abolished the HDAC3–DAD interaction, ablated the enzymatic activity of HDAC3 without altering its protein levels (You et al., 2013). NS-DADm mice are viable while whole-body HDAC3 knockout is embryonic lethal due to developmental defects (Bhaskara et al., 2008; You et al., 2013). This suggests that HDAC3 protein, but not its enzymatic activity, is required for normal development. Depletion of HDAC3 in the liver of adult mice causes remarkable hepatic steatosis, which can be rescued by wild-type (WT) or catalytically inactive mutants of HDAC3, demonstrating that the function of HDAC3 in hepatic lipid metabolism does not require its enzymatic activity (Sun et al., 2013). In addition to catalyzing the deacetylation reactions, HDAC3 can serve as a chaperone or a scaffold anchorage protein that regulates chromatin accessibility or corepressor complex dynamics, which can underlie its enzymatic-independent function (Gupta et al., 2009; Sun et al., 2013; Janardhan et al., 2017; Poleshko et al., 2017). These findings warrant further studies to address whether the physiological function of HDAC3 is dependent on its enzymatic activity in each tissue.

We recently showed that mice with HDAC3 specifically depleted in skeletal muscle (HDAC3-SkMKO) display reduced glucose utilization and reciprocally enhanced lipid oxidation (Hong et al., 2017; Gong et al., 2018). This metabolic change leads to reduced muscle force but enhanced fatigue resistance (Hong et al., 2017). Further studies revealed that HDAC3 depletion upregulated expression of many genes involved in branched-chain amino acids (BCAAs) catabolism, including AMP deaminase 3 (Ampd3), a rate-limiting enzyme in the purine nucleotide cycle that connects BCAAs catabolism with anaplerosis (Hong et al., 2017; Gong et al., 2018). Thus, the enhanced BCAAs catabolism boosts the oxidative capacity and contributes to the altered fuel metabolism in HDAC3-depleted muscles. Consistent with the enhanced BCAAs catabolism and the associated proteolysis, HDAC3-depleted muscle displayed reduced muscle mass during aging (Hong et al., 2017). Here we use the NS-DADm mice to address whether the muscular function of HDAC3 is dependent on its enzymatic activity. We found that abolishing the enzymatic activity of HDAC3 without affecting its protein levels is sufficient to replicate many metabolic phenotypes as previously observed in the HDAC3-depleted muscles. These findings suggest that, unlike in development or liver, functions of HDAC3 in skeletal muscles require its enzymatic activity.

## Results

### NS-DADm abolishes the enzymatic activity of HDAC3

**Figure 1:**
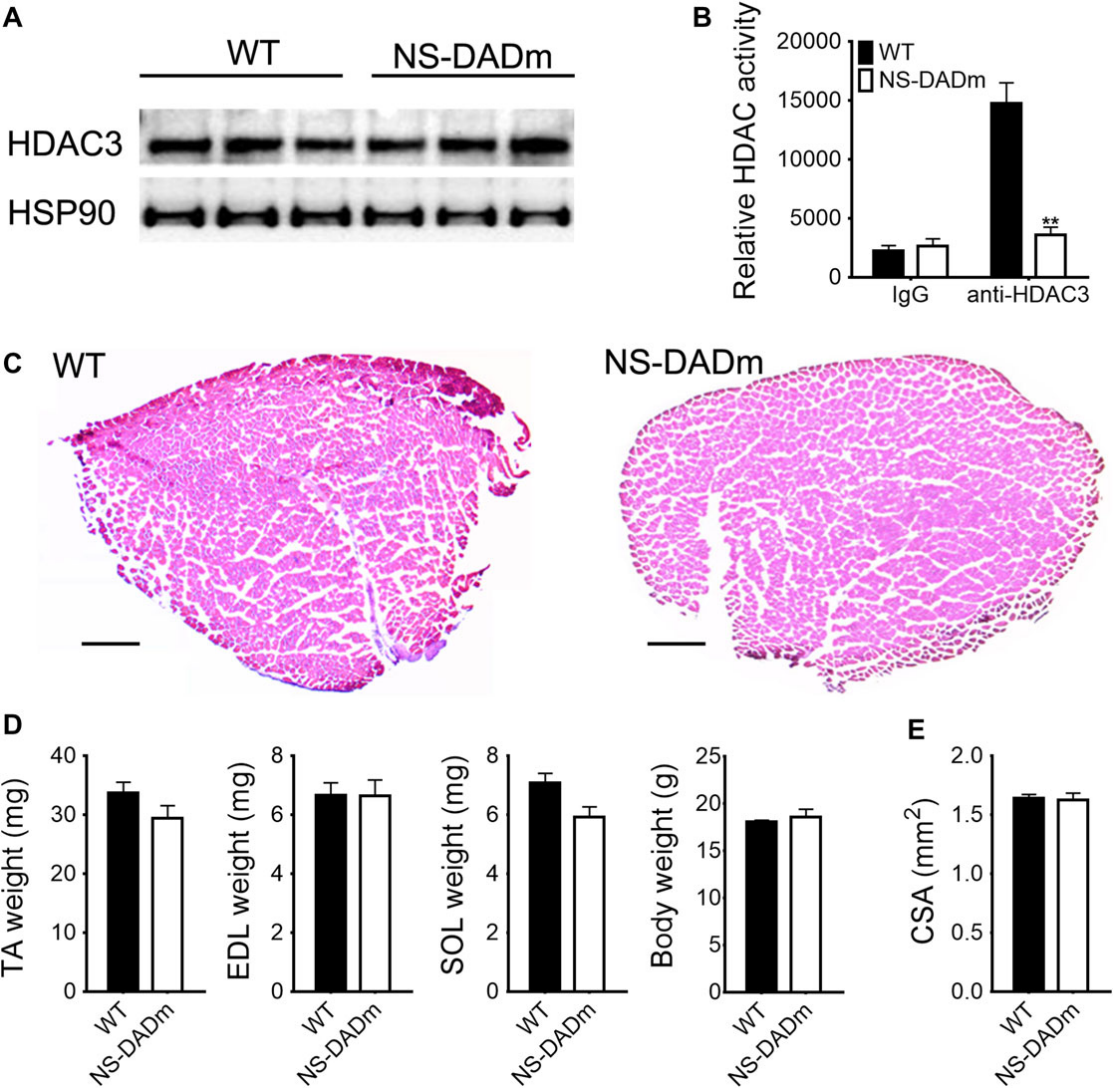
NS-DADm abolishes the enzymatic activity of HDAC3 in muscles. (**A**) Western blot analysis of tibialis anterior (TA) muscles from 4-month-old WT and NS-DADm mice. (**B**) Fluorescence-based HDAC enzyme assay using anti-HDAC3 immunoprecipitates from quadriceps muscles of WT and NS-DADm (*n* = 3 mice). (**C**) H&E staining of TA muscles from 5-month-old WT and NS-DADm mice. Scale bar, 600 μm. (**D**) Muscle mass and body weight of WT and NS-DADm mice at age of 2 months (*n* = 5 mice). SOL, soleus. (**E**) Muscle CSA calculated from length and weight of the EDL muscles dissected from 4-month-old mice (*n* = 5 mice). Data are presented as mean ± SEM. **P < 0.01 between genotypes with unpaired t-test.

NS-DADm mice were generated by crossbreeding N-DADm (NCoR-Y478A) mice with S-DADm (SMRT-Y470A) knock-in mice (Sun et al., 2013). Both mouse lines are based on C57BL/6 J background, and the age-matched wild-type (WT) C57BL/6 J were used as the control. NS-DADm mice were born at Mendelian ratios and did not show obvious developmental defects (Sun et al., 2013). Western blot analysis showed unaltered HDAC3 protein levels in skeletal muscles in adult NS-DADm mice (Figure 1A). The enzymatic activity of HDAC3 was measured using a fluorescence-based assay after HDAC3 was immunoprecipitated from the quadriceps muscles protein lysates, and was found efficiently abolished in NS-DADm muscles compared to the WT muscles (Figure 1B). NS-DADm mice displayed normal muscle morphological characteristics in H&E staining (Figure 1C). The muscle mass of NS-DADm mice was comparable with WT mice at 2 months old (Figure 1D). The cross-sectional area (CSA) was calculated from length and weight of the dissected extensor digitorum longus (EDL) muscles, and was comparable between WT and NS-DADm mice (Figure 1E). These results demonstrate that NS-DADm mutations abolished the enzymatic activity of HDAC3 in muscles, without affecting HDAC3 protein levels or the overall muscle morphology.

### Fiber-type composition in NS-DADm muscles

**Figure 2:**
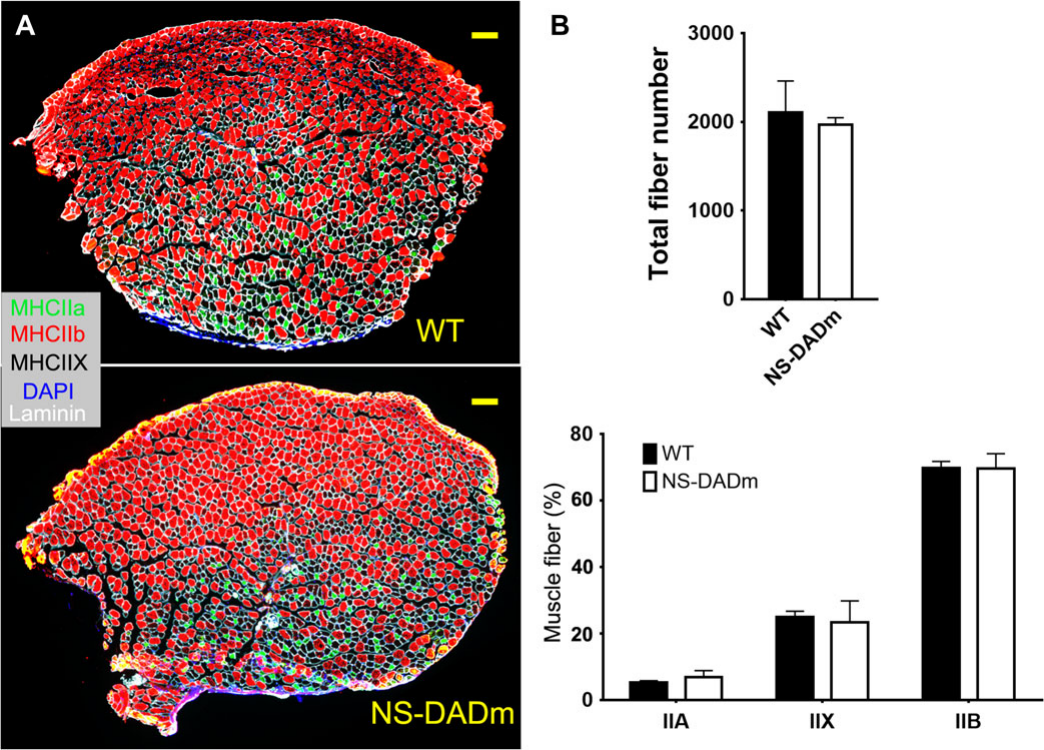
Fiber composition of NS-DADm muscles. (**A**) Representative immunofluorescence staining of cross-sections of TA muscles from 5-month-old WT and NS-DADm mice. Scale bar, 200 μm. (**B**) Quantification of the immunofluorescence staining results. Numbers for each type of fibers were counted from a cross-section of each individual mouse (*n* = 3 mice). Data are presented as mean ± SEM.

Skeletal muscles are composed of slow-contracting fatigue-resistant oxidative fibers (type I), fast-contracting fatigue-prone glycolytic fibers (type IIB), and intermediate fibers (type IIA and IIX). Expression of specific myosin heavy chain (MHC) isoforms is the defining characteristics for different fiber types (Zierath and Hawley, 2004; Schiaffino and Reggiani, 2011; Gan et al., 2013). We analyzed the fiber-type composition in NS-DADm TA muscles at the age of 5 months using immunofluorescence staining with MHC isoform-specific antibodies (Figure 2A). We did not show type I fibers because TA muscles of C57BL/6 mice contain almost no type I fibers (Kammoun et al., 2014). The total fiber number and the fiber-type composition are comparable between NS-DADm and WT mice (Figure 2B). These findings further support normal muscle development in NS-DADm mice and are in line with the unaltered fiber-type composition in HDAC3-depleted skeletal muscles (Hong et al., 2017).

### Contractile physiology of NS-DADm skeletal muscles

**Figure 3:**
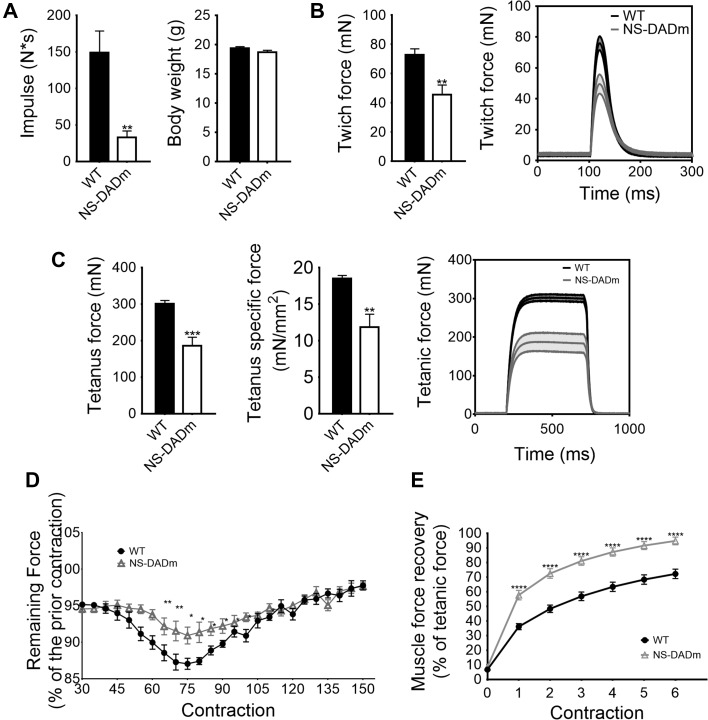
Contractile physiology of NS-DADm skeletal muscles. (**A**) Four-limb hanging test of 2-month-old WT and NS-DADm mice (*n* = 6–8). Holding impulse was calculated as the longest hanging time multiplied by the body weight. (**B **and** C**) Muscle force generation in EDL muscles isolated from 4-month-old female mice in *ex vivo* twitch contractions or tetanus contractions (*n* = 4 mice). The specific force was calculated by dividing the tetanic absolute force with muscle CSA. Average traces of the *ex vivo* twitch and tetanic contractions were shown with the shaded area as SEM. (**D**) Muscle fatigue was induced by repetitive stimulation. The fatigue index was expressed as the percentage of force left for every five contractions. (**E**) Force recovery after exhaustion (*n* = 4 mice). Data are presented as mean ± SEM. *P < 0.05, **P < 0.01, ***P < 0.001, ****P < 0.0001 between genotypes by unpaired *t*-test.

To characterize the muscle contractile function, we performed a four-limb hanging test. The holding impulse (body mass × hang time) is a measure of the ability to produce sustained tension in the limb musculature against gravity. NS-DADm mice showed a lower holding impulse than WT mice (Figure 3A). This suggests a possible reduction in muscle strength, although hormonal and neurophysiological factors in this whole-body knock-in mice could contribute to the phenotype. To exclude these extra-muscular factors, we performed *ex vivo* contractile physiology analysis using isolated EDL muscles from 4-month-old NS-DADm mice and WT mice. NS-DADm muscles generated less twitch force and tetanus force than WT (Figure 3B and C). To test muscle fatigability, EDL muscles were subjected to repeated field electric stimulations with continuous force measurement. The rate of force dropping was presented as the percentage of remaining force of a previous contraction detected once every five contractions. NS-DADm muscles demonstrated a higher force retaining ability than WT, suggesting enhanced fatigue resistance (Figure 3D). NS-DADm muscles also showed a quicker recovery from exhaustion than WT (Figure 3E). A similar change in force generation and fatigability was observed in male mice, suggesting that the phenotype is not dependent on sex (Supplementary Figure S1). The reduced muscle strength and enhanced fatigue resistance in NS-DADm muscles are similar to what was previously observed in the *ex vivo* physiology study with HDAC3-depleted muscles (Hong et al., 2017), which suggests that the HDAC3 enzymatic activity is required for its function in muscle contraction.

### Altered fuel metabolism in NS-DADm muscles

**Figure 4:**
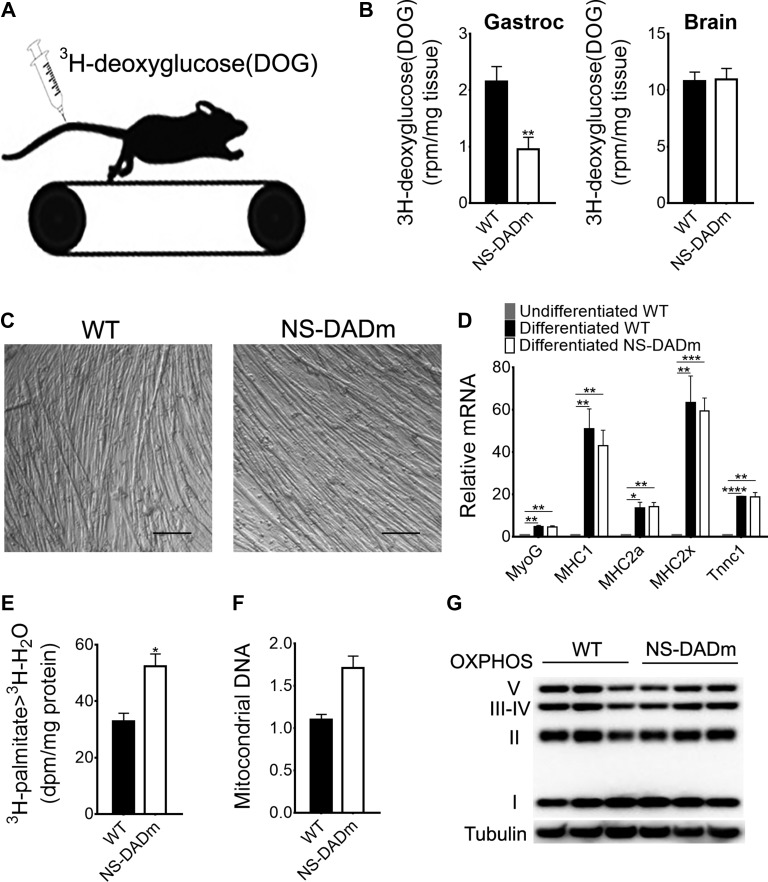
Altered fuel metabolism in NS-DADm muscles. (**A**) Mice were injected with ^3^H-deoxyglucose (DOG) through tail vein immediately before treadmill running. (**B**) Tissue-specific DOG uptake in gastrocnemius (gastroc) muscles and brains of 4-month old mice (*n* = 5 mice). (**C**) Representative images of differentiated myotubes from isolated primary myoblasts. (**D**) RT-qPCR analysis of differentiation markers (*n* = 3 wells of cells). (**E**) Fatty acid oxidation in primary myotubes (*n* = 3 wells of cells). (**F**) Mitochondrial DNA quantification by qPCR in whole-genome extraction of TA muscles. Mitochondrial gene (mtND1) was normalized to a nuclear gene Ndufv1 (*n* = 4 mice). (**G**) Western blot analysis of mitochondrial OXPHOS complexes in TA muscles of 5-month-old mice (*n* = 3 mice). Data are presented as mean ± SEM. *P < 0.05, **P < 0.01, ***P < 0.001, ****P < 0.0001 between genotypes by unpaired *t*-test.

Muscle fuel metabolism and bioenergetics are important for contractile function. Reduced glucose utilization and enhanced fatty acid oxidation contribute to the contractile phenotype in the HDAC3-depleted muscles (Hong et al., 2017). NS-DADm mice showed normal blood insulin levels at the baseline and displayed similar responses to injected insulin during an insulin tolerance test (Supplementary Figure S2). We used ^3^H-deoxyglucose as a metabolic tracer to assess *in vivo* muscle-specific glucose uptake during exercise in NS-DADm mice. Mice were subjected to treadmill running for 20 min, followed by i.v. injection of ^3^H-deoxyglucose. Mice were then allowed to run for an additional 35 min before tissue harvest, followed by processing and scintillation counting (Figure 4A). Muscle-specific glucose uptake was significantly lower in NS-DADm mice than WT mice, while unaltered brain glucose uptake served as a negative control (Figure 4B). These data demonstrated a reduced utilization of glucose in skeletal muscles of NS-DADm mice during exercise. In addition to glucose, fatty acids are another major fuel for muscle contraction. To assess muscle-specific lipid oxidation, we used ^3^H-palmitate as a tracer in primary myotubes. Primary myoblasts isolated from NS-DADm and WT mice were allowed to differentiate into myotubes. Efficient differentiation was confirmed by microscopic examination of myotubes formation (Figure 4C) and robust upregulation of differentiation marker genes (Figure 4D). Myotubes from both WT and NS-DADm were able to contract upon pacing with electric pulse stimulation (EPS), suggesting that NS-DADm mutations did not affect the myogenic process or myotube differentiation. Compared to WT, the NS-DADm myotubes were more efficient in converting ^3^H-palmitate to ^3^H-H_2_O, demonstrating enhanced fatty acid oxidation (Figure 4E). This is in keeping with the enhanced fatigue resistance considering that lipid carries more calories per mass unit and is a better fuel for endurance exercise. Mitochondrial DNA copy number was increased in NS-DADm muscles (Figure 4F), but the oxidative phosphorylation (OXPHOS) protein content was unaltered (Figure 4G), suggesting that changes in the oxidative metabolic flux, rather than mitochondrial content, contribute to the altered fuel metabolism. Taken together, NS-DADm muscles have reduced glucose utilization but enhanced lipid oxidative metabolism during contraction without changing total mitochondrial OXPHOS protein content, which is similar to what was previously observed in HDAC3-depleted muscles (Hong et al., 2017).

### Molecular changes in NS-DADm muscles

**Figure 5:**
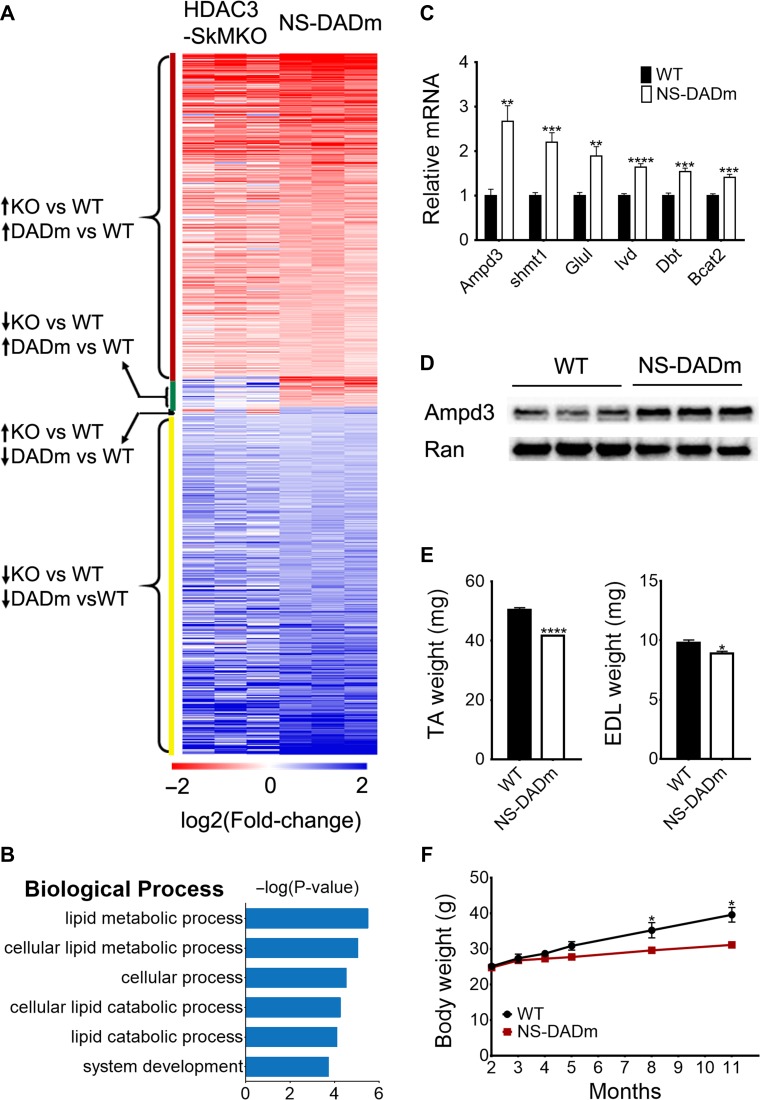
Molecular changes in NS-DADm muscles. (**A**) Heat map comparison of RNA-seq analyses of NS-DADm muscles and HDAC3-depleted muscles (SkMKO). Fold-change refers to the ratio of gene expression level in each individual NS-DADm or HDAC3-SkMKO mouse to the average of their respective WT controls. (**B**) Top enriched pathways of differentially expressed genes between NS-DADm muscles and WT controls. (**C**) RT-qPCR analysis of gene expression in TA muscles from WT and NS-DADm mice (*n* = 6). (**D**) Western blot analysis of TA muscles from 5-month-old WT and NS-DADm mice. Ran served as the loading control. (**E**) Muscle mass of 8-month-old WT and NS-DADm mice (*n* = 4). (**F**) Body weight of WT and NS-DADm mice (*n* = 6–9). Data are presented as mean ± SEM. **P < 0.01, ***P < 0.001, ****P < 0.0001 between genotypes by unpaired *t*-test.

We performed RNA-seq analysis to address how gene expression is regulated by the HDAC3 enzymatic activity in muscles. In our previous study of HDAC3 muscle-specific knockout mice (HDAC3-SkMKO), we found that HDAC3 depletion upregulates BCAAs catabolism, which accounted for the increased fatty acid oxidation through anaplerotic reactions (Hong et al., 2017). Here we found that NS-DADm muscles displayed a similar gene expression pattern as HDAC3-SkMKO (Figure 5A). Compared to their respective WT controls, >300 genes were upregulated in both NS-DADm muscles and HDAC3-depleted muscles. Conversely, >300 genes were downregulated in both NS-DADm muscles and HDAC3-depleted muscles. In comparison, only 35 genes were changed in the opposite directions in NS-DADm muscles and HDAC3-depleted muscles (Figure 5A). Differentially expressed genes between NS-DADm and WT muscles were highly enriched in BCAAs catabolism (Figure 5B), which is similar to what was previously observed in HDAC3-depleted muscles (Hong et al., 2017). RT-qPCR analysis further validated the upregulated expression of several key genes involved in BCAAs catabolism (Figure 5C). AMP deaminase 3 (Ampd3), a rate-limiting enzyme in the purine nucleotide cycle, was upregulated at the protein level in NS-DADm muscles compared to the WT control (Figure 5D).

The purine nucleotide cycle is a major anaplerotic reaction in skeletal muscles. We have shown that Ampd3 overexpression enhanced amino acid catabolism and fatty acid oxidation in myotubes, which contributes to the altered fuel metabolism in the HDAC3-depleted muscles (Hong et al., 2017). Although HDAC3-SkMKO mice are able to maintain normal muscle mass at a young age, the enhanced BCAAs catabolism and associated muscle proteolysis eventually led to reduced muscle mass when HDAC3-SkMKO mice grow old (Hong et al., 2017). Here we found that NS-DADm mice showed a similar aging-related lower muscle mass and body weight compared to control mice (Figure 5E and F). Of note, we tested muscle performance at a young age to avoid confounding effects of muscle mass difference in both NS-DADm mice and HDAC3-SkMKO mice. The metabolic change, rather than the mass reduction, is likely the primary cause of the altered muscle performance in the NS-DADm muscles, since they showed lower force generation even before they showed reduced muscle mass. This is similar to HDAC3-SkMKO muscles and suggests that the enzymatic activity of HDAC3 plays an important role in the process.

## Discussion

We hereby address how the HDAC3 enzymatic activity *per se* regulates muscle fuel metabolism. Using the NS-DADm mouse model, we ablated the enzymatic activity of HDAC3 *in vivo* without affecting its protein levels and compared this mouse model with our previous skeletal muscle-specific HDAC3 knockout (HDAC3-SkMKO) mouse model (Hong et al., 2017). A potential caveat of comparing the two mouse models is that changes in other tissues in the whole-body knock-in NS-DADm mice could indirectly affect the muscle phenotype through circulating or neural factors. To minimize these potential confounding factors, we not only characterized the *in vivo* muscle phenotype but also performed a series of *ex vivo* analyses using muscles or primary myocytes isolated from NS-DADm mice. Compared to WT mice, skeletal muscles from NS-DADm mice showed lower force generation, enhanced fatigue resistance, enhanced fatty acid oxidation rate, lower glucose uptake during exercise, upregulated expression of metabolic genes involved in branched-chain amino acids (BCAAs) catabolism, and aging-associated reduction in muscle mass, without changes in the muscle fiber-type composition or mitochondrial protein content. All these muscle phenotypes are similar to those previously observed in HDAC3-SkMKO mice (Hong et al., 2017; Gong et al., 2018). Collectively, these data demonstrate that the metabolic function of HDAC3 in the skeletal muscle requires its enzymatic activity.

Proteins are versatile, and many enzymes can have enzyme-independent functions (Eyers and Murphy, 2016). Class IIa HDACs (HDAC4, HDAC5, HDAC7, and HDAC9) lack intrinsic deacetylase activities due to a tyrosine-to-histidine substitution at the catalytic pocket (Lahm et al., 2007). Their enzymatic activity is dependent on HDAC3 that is often found in the same corepressor complex as class IIa HDACs (Fischle et al., 2002). Therefore, it is believed that class IIa HDACs might work as a chaperone to bring HDAC3 to its associated substrate. We and others showed that HDAC3 also has enzyme-independent functions in cardiovascular development and hepatic lipid metabolism (Gupta et al., 2009; Sun et al., 2013; Lewandowski et al., 2015; Janardhan et al., 2017; Poleshko et al., 2017). Given that many small molecules target HDACs enzymatic activity but not their protein levels, it is important to examine on a case-by-case basis to what degree each HDAC requires its enzymatic activity for their physiological functions. In line with the current study, the HDAC3 enzymatic activity was found to be required for productive VDJ recombination in B lymphocytes (Stengel et al., 2017). These findings suggest that the functional dependency of HDAC3 on its enzymatic activity is context-dependent and tissue-specific.

A potential limitation of our study is that the NS-DADm could affect other unknown functions of NCoR/SMRT in addition to abolishing the DAD-HDAC3 interaction, although the possibility is low considering the following facts: (i) DAD is a small domain (~90 amino acids) in a large protein (~2500 amino acids) and is not required for interaction with most known NCoR/SMRT-binding proteins except for HDAC3 and p53 (Wong et al., 2014). (ii) NS-DADm are missense mutations (NCoR-Y478A/SMRT-Y470A) specifically required for HDAC3 enzyme activity. Y470A does not affect SMRT interaction with p53 or the ability of SMRT to co-activate p53-dependent gene expression (Adikesavan et al., 2014). (iii) NS-DADm mutations affect gene transcription and biological phenotypes in a much milder manner compared to NCoR/SMRT knockout in different tissues or developmental stages. For example, NCoR or SMRT whole-body knockout mice are embryonic lethal (Jepsen et al., 2000, 2007) while N-DADm or NS-DADm mice survive to adulthood without obvious developmental defects (Alenghat et al., 2008; You et al., 2013). Muscle-specific knockout of NCoR altered fiber-type composition and expression of genes in oxidative phosphorylation (Yamamoto et al., 2011; Pérez-Schindler et al., 2012), while muscles from NS-DADm mice did not show such changes in our current study. These findings suggest that the general function of the NCOR/SMRT remains unaltered in the NS-DADm mouse model.

HDAC3 is considered a major target for therapeutic benefits of many HDIs in cancer, immune diseases, metabolic disorders, and neurological diseases (Cao et al., 2018). The establishment of HDAC3 enzymatic activity in regulating muscle fuel metabolism raised the possibility of using HDIs to manipulate muscle fuel preference or enhance exercise performance. Boosting muscle lipid oxidation can be desirable for weight loss or enhancing exercise endurance. Low-carbohydrate high-fat ketogenic diets were tested for these purposes, with some success (Chang et al., 2017; Abbasi, 2018; McSwiney et al., 2018). Combination of ketogenic diets with HDIs may provide a more desirable outcome than diets alone. However, potential side effects of HDIs on muscles should be noted, including reduced muscle strength or impaired muscle insulin sensitivity. The latter is related to the mutual competition between burning glucose and burning lipids in muscles (RANDLE et al., 1963; Kelley and Mandarino, 2000; Muoio, 2014). Muscle insulin sensitivity in terms of glucose utilization is negatively correlated with muscle lipid oxidation, as recently shown in several genetic mouse models of transcriptional regulators in addition to HDAC3 (Lin et al., 2002; Finck et al., 2005; Calvo et al., 2008; Choi et al., 2008; Wong et al., 2015; Meng et al., 2018). Several transcription factors or cofactors associated with HDAC3 have also been shown to regulate muscle performance and metabolism (Yamamoto et al., 2011; Pérez-Schindler et al., 2012; Woldt et al., 2013). Pharmacological targeting of these factors in combination with HDIs could be potentially developed and tested for remodeling muscle fuel metabolism to benefit weight loss or enhance exercise performance.

## Methods

### Mouse

NS-DADm mice were generated from crossing N-DADm and S-DADm (You et al., 2013). All mice were based on the C57BL/6 J background. Mice were housed under a 12-h light/12-h dark cycle (lights on at 7 a.m. and lights off at 7 p.m.). Male mice at the age of 2–8 months were used for all experiments unless otherwise indicated. Tissues were harvested at 4–5 p.m. without restricting the mice to food and water unless indicated otherwise in figure legends. All the animal care and use procedures followed the guidelines of the Institutional Animal Care and Use Committee of the University of Pennsylvania and Baylor College of Medicine.

### Western blot and HDAC assay

For western blot, tissues or cells were lysed in RIPA buffer, which contained PMSF and proteinase inhibitor. The concentration of total protein was quantified using Bradford protein assay. Lysates were resolved on SDS-PAGE gel electrophoresis and then transferred to PVDF membranes. The membranes were further treated with skimmed milk and blotted with antibodies of HDAC3 (Abcam 7030), Ampd3 (Abcam 194361), Hsp90 (CST 4874), Tubulin (CST 2148), and OXPHOS proteins (MitoSciences MS604). For HDAC assay, the HDAC3 protein was immunoprecipitated from the muscle protein lysates with 2 mg total protein, followed by a fluorescence-based HDAC assay reaction (Active Motif) performed with the pelleted beads (Sun et al., 2013).

### Histology and immunofluorescence staining

Fresh tibialis anterior (TA) muscles were isolated, embedded in OCT compound, and frozen in the dry ice chilled isopentane. Cross-sections at 10-μm thickness were obtained using a Leica CM1850 cryostat. Hematoxylin and eosin (H&E)-stained images were captured with a Leica inverted microscope. For immunofluorescence staining, tissue sections were incubated with mixed primary antibodies: laminin (Sigma), MHC-IIa (2F7; Developmental Studies Hybridoma Bank), and MHC-IIb (BF-F3; Deutsche Sammlung von Mikroorganismen und Zellkulturen) in blocking buffer, followed by incubation with secondary antibodies: Alexa-647-goat anti-rabbit, Alexa-594-goat anti-mouse IgG, and Alexa-488-goat anti-mouse IgM (A32733, A21121, A21426; Invitrogen, dilution: 1:1000 in PBS) (Waddell et al., 2010). Unstained fibers were counted as MHC-IIX, based on the fact that mouse TA muscles contain few type I fibers (Augusto et al., 2004). Fluorescent images were captured with Zeiss Axiophot epifluorescence microscope and Axiovision software. The CSA was calculated from length and weight of the dissected EDL muscles.

### Wire hang test and muscle contractile physiology

For four-limb wire hang test, mice were placed on a grid where it stood using all four limbs. The grid was then turned upside down 30 cm above a cage. Mice that fell down before the 10 min time limit were given two more tries. The longest hanging time was used for calculating the holding impulse (the hang time multiplied by the body weight [conversion factor 9.8 Newtons/kg]) (Carlson et al., 2010). Muscle physiological analysis was performed on isolated EDL muscles using an Aurora Mouse 1200 A System equipped with Dynamic Muscle Control v.5.415 software. EDL muscles were dissected and analyzed in constantly oxygenated Ringer’s solution (100 mM NaCl, 4.7 mM KCl, 3.4 mM CaCl_2_, 1.2 mM KH_2_PO_4_, 1.2 mM MgSO_4_, 25 mM HEPES, 5.5 mM D-glucose, and 2% BSA) at 24°C. The twitch stimulation protocol applied was a single stimulus with a duration of 0.2 ms. For measuring tetanic maximal force generation, the stimulus was repeated at a frequency of 120 Hz for 500 ms. Five minutes were allowed between two tetanic contractions to ensure muscle recovery. For induction of fatigue, 5 min after the last maximal tetanic contraction, muscles were stimulated every second for 8 min using 40-Hz pulses lasting 330 ms (Alenghat et al., 2008). The fatigue index was expressed as the percentage of force left after five contractions. Following the fatigue protocol, a burst of 50 maximal tetanic contractions (120 Hz for 500 ms) was applied to ensure complete fatigue across all samples. The recovery protocol started 1 s after the last burst contraction. A maximal tetanic stimulation (120 Hz for 500 ms) was given every 5 min for 30 min, and the force recovery was expressed as the percentage of the maximal isometric tetanic force.

### RNA-seq and RT-qPCR

RNA-seq and RT-qPCR were performed using total RNA extracted from TA muscles (*n* = 3). Total mRNA was extracted with TRIzol reagent followed by RNeasy mini columns (Qiagen). For RT-qPCR, a Thermo NanoDrop One was used to assess the concentration and quality of RNA before reversed transcription and amplification. For RNA-seq data processing, sequenced reads from six mice (three control mice and three NS-DADm mice) were aligned to the mm10 mouse genome (GRCm38) using STAR aligner (v2.5.3a) with default parameters. Differential gene analysis was carried out using DESeq2 package in R environment (Love et al., 2014). Significantly differentially expressed genes were identified based on 5% false discovery rate threshold. Gene ontology was performed using DAVID Bioinformatics Resources 6.7, and the pathway enrichment was performed on KEGG pathways.

### Primary myoblasts isolation and differentiation

Primary myoblasts were isolated from hindlimb skeletal muscles. Muscles were minced and digested in type I collagenase and Dispase B mixture (Roche Applied Science). Cells were filtered from debris, centrifuged, and cultured in growth medium (F-10 Ham’s medium supplemented with 20% FBS, 5 ng/ml basic fibroblast growth factor, and 1% penicillin-streptomycin) on collagen-coated cell culture plates at 37°C, 5% CO_2_. Multiple pre-plating was used to remove fibroblasts. Growth medium was changed every 2 days (Wen et al., 2012). When the primary myoblasts get confluent, growth medium was changed to differentiation medium (DMEM supplemented with 2% fetal horse serum and 1% penicillin-streptomycin). Differentiated myotubes and myogenic cells were harvested for further investigations in 7 days, and fresh differentiation medium was added every 2 days.

### Isotope tracing in vitro

In order to detect the fatty acids oxidation, differentiated myotubes were incubated in PBS, and supplemented with [9,10-3H(N)]-palmitate, conjugated on BSA and carnitine for 120 min. EPS using the IonOptix C-pace system with 15 volts at 1 Hz was applied 30 min before adding the [9,10-3H(N)]-palmitate. The resultant ^3^H-H_2_O in the incubation solution was separated from precursors using ion-exchange columns (DOWEX 1X4-400), and was measured by scintillation counter.

### Glucose uptake in vivo

Mice were subjected to treadmill running for 20 min, followed by i.v. injection of 500 μCi/kg ^3^H-2-deoxy-D-glucose (2DG). Subsequently, mice ran on the treadmill at 10 m/min for 35 min before muscles were harvested and frozen in liquid nitrogen. The 2DG6P contents in tissues were measured and were normalized to the tissue mass (Stenbit et al., 1996).

### Statistics

Student’s two-tailed *t*-test was performed to determine significant differences between genotype groups for all experiments except the RNA-seq experiment. Statistics and SEM were calculated using an individual mouse as one sample for *in vivo* or *ex vivo* experiments and an individual well of the cultured cell as one sample for *in vitro* experiments. No sample was excluded. Fiber quantification, *ex vivo* muscle contraction physiology, and RNA-seq were performed by experimenters who were blinded to the mouse genotype.

### Data accession

RNA-seq data were uploaded to Gene Expression Omnibus (GEO) with accession #GSE115226.

## Supplementary Material

Supplementary Data
