# Supplementary material for: The HDAC3 enzymatic activity regulates skeletal muscle fuel metabolism
Source: J Mol Cell Biol. 2018 Nov 14;11(2):133–43. doi: 10.1093/jmcb/mjy066 (PMC6392100; doi:10.1093/jmcb/mjy066)
Supplement: Supplementary Data [file mjy066_supplementary_material.zip › mjy066_Supplementary_material.pdf]

Supplementary material

The HDAC3 enzymatic activity regulates skeletal muscle fuel metabolism

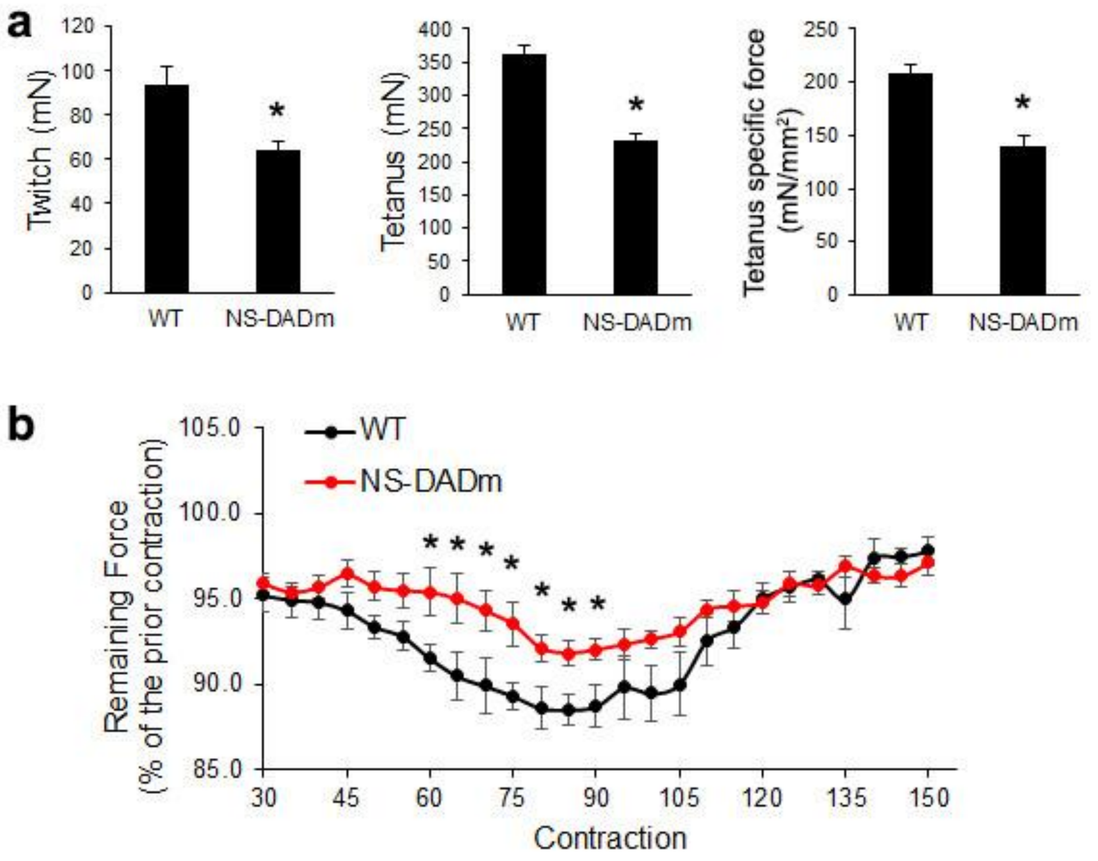

Supplementary Figure S1. *Ex vivo* muscle physiology analysis in male mice. (a) Muscle force generation in extensor digitorum longus (EDL) muscles isolated from 4-month old male mice in *ex vivo* twitch contractions or tetanus contractions (n = 4 mice). The specific force was calculated by dividing the tetanic absolute force with muscle cross-sectional area. **(b)** Muscle fatigue was induced by repetitive stimulation. The fatigue index was expressed as the percentage of force left for every 5 contractions. Data were presented as the mean ± S.E.M. * $P<0.05$ between genotypes by unpaired t-test.

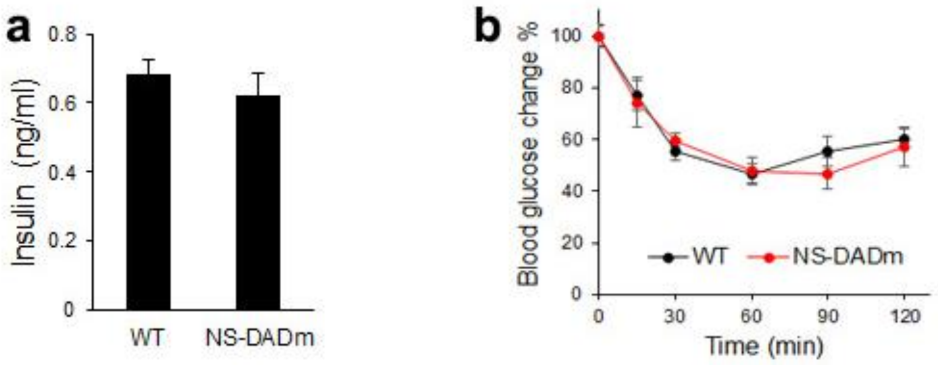

Supplementary Figure S2. Metabolic characterizations of NS-DADm mice.
(a) Fasting blood insulin levels in 4-month old female NS-DADm mice (n = 6-7). **(b)** Insulin tolerance test. Insulin was i.p. injected at 0.75U/kg and blood glucose was measured at the indicated time after insulin injection (n = 6-7). Data were presented as the mean ± S.E.M.
